# A disorder-specific group cognitive behavior therapy for social anxiety disorder in adolescents: study protocol for a randomized controlled study

**DOI:** 10.1186/s13063-019-3885-3

**Published:** 2019-12-21

**Authors:** Nanna Fensman Lassen, Esben Hougaard, Kristian Bech Arendt, Mikael Thastum

**Affiliations:** Department of Psychology and Behavioural Sciences, Aarhus University, Aarhus, Denmark

**Keywords:** Anxiety, Social anxiety disorder, Cognitive behavior therapy, Randomized controlled trial, Adolescents

## Abstract

**Background:**

Social anxiety disorder (SAD) is a common disorder in adolescence associated with extensive distress and long-term impairment. Generic cognitive behavior therapy (CBT) programs for anxiety disorders have shown poorer outcomes for adolescents with SAD than for other anxiety disorders.

**Aim:**

The aim of the present study is to investigate the efficacy of a disorder-specific group cognitive behavior therapy (G-CBT) program for youth SAD, the Cool Kids Anxiety Program - Social Enhanced (CK-E), developed at Macquarie University, Sidney, Australia.

**Methods:**

The study is a randomized controlled trial comparing CK-E to a generic G-CBT program for anxiety disorders. Approximately 96 adolescents aged 12 to 17 years are included with data points at pre- and post-treatment, and at 3 months and 1 year follow-ups.

**Discussion:**

The current study will provide more information about the efficacy of diagnosis-specific G-CBT treatment for youth SAD.

**Trial registration:**

ClinicalTrials.gov, NCT03986827. Registered on 14 June 2019.

## Background

Social anxiety disorder (SAD) most often starts in the early teens with a median age of onset at 12.1 according to the adult sample of the National Comorbidity Survey Replication in the USA [29]. It is among the most common of anxiety disorders, with 5–9% of adolescents between 13 and 18 years suffering from SAD [10, 16, 29]. Furthermore, studies have shown an increase in SAD from childhood through adolescence [8, 10, 12].

If left untreated, SAD increases the likelihood of chronicity, loneliness, problems in relation to school activities, and risk of other anxiety disorders, depression, and substance abuse [7, 8, 10, 30, 47]. Adolescence can be regarded as a critical period in the treatment of SAD in order to avoid a chronic developmental course [39, 56]. Thus, development of effective treatment for adolescents suffering from SAD is crucial.

Cognitive behavior therapy (CBT) is the best-documented treatment for anxiety disorders in youths [28]. Generic CBT programs for children and adolescents struggling with anxiety disorders have shown substantial effects for other anxiety disorders [5, 46]. However, recent studies indicate that adolescents with SAD have poorer outcomes following generic treatment compared to adolescents struggling with other anxiety disorders like generalized anxiety, separation anxiety, specific phobia, or obsessive compulsive disorder [26, 31, 33, 46].

Different CBT programs specifically designed to treat adolescents with SAD have been developed [9, 24, 25, 37], but there are few direct comparisons of diagnosis-specific treatment of SAD in youth with generic CBT treatment programs [39]. Ingul, Aune and Nordahl [27] compared a diagnosis-specific individual CBT (I-CBT) program for youth SAD with generic G-CBT and found diagnosis-specific treatment more effective than generic. However, the different format of the two treatments limits the conclusion as to the role of specific treatment ingredients. Besides, generic G-CBT achieved no change from pre- to post-treatment, which raises doubt about the quality of this treatment.

There is considerable evidence for diagnosis-specific individual CBT for SAD among adults [36, 39]. There is, however, no evidence of better results of individual CBT for SAD among children and adolescents [39, 64]. In their meta-analysis, Yang et al. [64] found comparable results for individual (*g* = 1.10) and group formats (*g* = 1.19) of psychological interventions for SAD in children and adolescents. They therefore suggested that the group format might be cost-effective in the treatment for youths with SAD. However, results from this meta-analysis should be interpreted with caution due to the high heterogeneity and low quality of most studies.

The present study compares a diagnosis-specific group CBT program for SAD with a generic group CBT program for mixed anxiety disorders. The diagnosis-specific intervention, the Cool Kids Anxiety Program - Social Enhanced (CK-E), was developed at the Centre for Emotional Health at Macquarie University, Sydney, Australia. The program is based on cognitive and behavioral processes that are theorized to maintain SAD [13, 44]. CBT including such strategies has been recommended for adults with SAD [39]. The generic program (CK) was essentially identical with the Macquarie Cool Kids Anxiety for adolescent anxiety disorders program (Chilled [45];), but with psychoeducation focusing on SAD. Both treatment programs were group-based with parent involvement.

A further mediational study based on the study data is planned in the future, but the present protocol focuses primarily on the randomized controlled trial (RCT).

### Aim and hypothesis

The aim of the study is to investigate the efficacy of a disorder-specific group CBT program for youth SAD (G-CK-E) compared to a generic group CBT program for anxiety disorders (G-CK). Reduction in anxiety symptoms is expected for both treatment conditions, although we hypothesize better outcome for the enhanced treatment condition.

## Methods

### Study design

The study will take place at the Centre for Psychological Treatment of Children and Adolescents (CEBU), a teaching and research facility at the Department of Psychology and Behavioral Sciences, Aarhus University, Denmark.

**Fig. 1:**
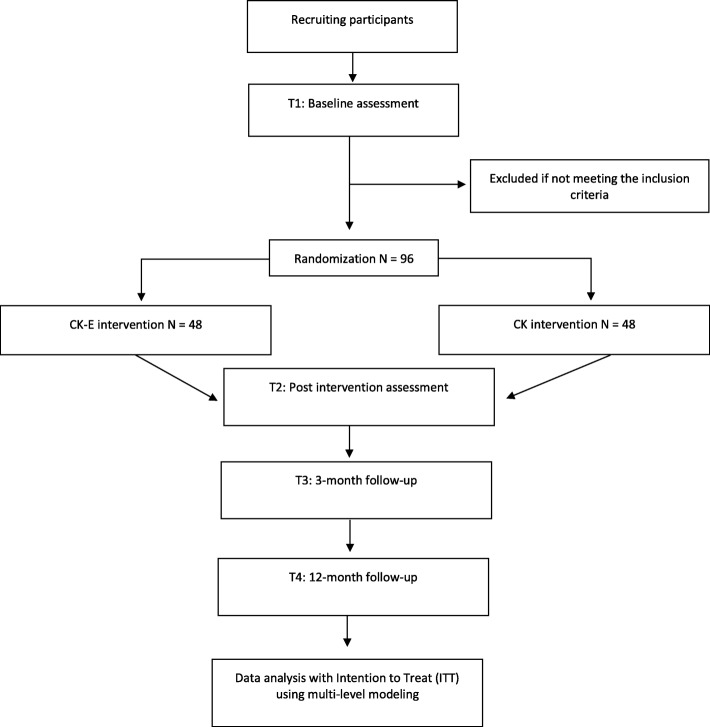
Flowchart of the study design

The study is a randomized controlled superiority trial comparing two active groups of treatment: (1) Cool Kids Anxiety Program - Social Enhanced (G-CK-E) and (2) Cool Kids Anxiety Program (G-CK). The study design is a mixed between-within design, with data points pre-treatment (T1) and post-treatment (T2), and at follow-ups of 3 and 12 months (T3 and T4). Figure 1 presents a flowchart of the study.

### Participants

Participants are adolescents between 12 and 17 years old who have SAD. Participants are self-referred, based on information on websites, newspaper advertisements, and hand-outs to local general practitioners and educational institutions. Interested families are invited to send a brief description of the adolescent’s major problems. An expected recruitment of 16 adolescents half-yearly will yield 96 adolescents completing treatment during a 3-year period.

Inclusion criteria are as follows: Participants must be (1) between 12 and 17 years of age; (2) have a SAD diagnosis as their primary disorder.

Exclusion criteria are the following: (1) a diagnosed autism spectrum disorder (ASD); (2) untreated attention deficit hyperactivity disorder (ADHD); (3) psychotic symptoms; (4) current severe self-harm or suicidal ideation; (5) current eating disorder; (6) Clinical Severity Rating (CSR) > 5 on depression (for clarification on CSR see Primary outcome measures); (7) received prior CK treatment within the last 2 years.

### Randomization

Randomization is stratified into two age groups (12–14 and 15–17) using a permuted block design with a fixed block size of 8 at a 1:1 ratio to the CK or the CK-E condition. Randomization is conducted with an online computer random number generator (www.random.org) by an independent secretary. The sequence list is kept concealed from therapists until treatment starts. Participants are not informed about their specific treatment condition.

### Intervention

Both treatment programs were translated from the original manualized, individual Australian CBT program and adjusted to fit a group format by staff at CEBU. Interventions in both treatment conditions consist of 10 2-h group sessions with four adolescents and their parents in each group. Sessions 1 through 8 are held weekly, and sessions 9 and 10 at 2-week intervals. Three months after ending treatment, participants are offered a 1-h booster group session. Table 1 presents an overview of the intervention programs.

**Table 1:** Overview of the intervention programs

Session number	Participants	Session content CK-E	Session content CK
S-1	T, Y, P	• Rapport building • Psychoeducation • Worry scale • SMART goals	• Psychoeducation • Fears list • Goals • Worry scale • Introduce linking thoughts and feelings
S-2	T, Y, P	• Continue rapport building • Introduce link between thoughts & feelings • Introduce attention training • Review parent goals	• Introduce cognitive restructuring (detective thinking) • Review parent goals
S-3	T, Y, P	• Introduce cognitive restructuring (detective thinking) • Introduce rewards	• Continue detective thinking • Introduce cool breathing • Introduce rewards
S-4	T, Y, P	• Detective thinking to cost • Introduce avoidance • Introduce behavioral experiments • Conduct in-session experiment	• Introduce exposure • Design 1st stepladder
S-5	T, P	• Parenting an adolescent with SAD	• Parenting an adolescent with SAD
S-6	T, Y, P	• Introduce safety traps • Review experiments • Introduce experiments to reduce safety behaviors • Conduct in-session experiment • Introduce task-focused attention	• Review exposure progress • Design further stepladders and review useful steps • Planning in-session exposure
S-7	T, Y, P	• Review safety trap experiments and task-focused attention • Conduct the video-feedback experiment • Introduce the importance of obtaining an accurate self-perception • Obtaining accurate self-perception using feedback • Conduct additional in-session experiments	• Review exposure progress • Simplified detective thinking (in my mind and act as if) • In-session exposure
S-8	T, Y, P	• Review and revise experiments that utilize feedback • Introduce post-event processing (detective thinking *after* a situation) • Introduce cost experiments • Conduct in-session exposure (including an extra challenge experiment)	• Review of exposure progress • In-session exposure • Problem solving
S-9	T, Y, P	• Review and revise extra challenge experiments and post-event detective thinking • In-session experiments • Parents only: Progress review, experiment revision, and (optional) experiment troubleshooting • Optional module: Dealing with teasing and bullying	• Revise/add stepladders • Conduct in-session exposure • Parents only: Progress review, stepladder revision, and (optional) stepladder practice troubleshooting
S-10	T, Y, P	• Review of goals • Maintenance of gains/setbacks • Future plans	• Review of goals • Maintenance of gains/setbacks • Future plans
Booster	T, Y,P	• Focusing on maintaining and continuing the progress • Advise possible further help	• Focusing on maintaining and continuing the progress • Advise possible further help

Abbreviations: *S* session, *Y* youth, *P* parent, *T* therapist, *CK-E* Cool Kids Anxiety Program - Social Enhanced, *CK* Cool Kids Anxiety Program

Four clinical psychologists from CEBU with experience in the Cool Kids programs (2–5 years) will conduct the treatment intervention. There is one therapist per treatment group, with three to four graduate students attending as practical assistants (helping with exercises and filling out scales).

The clinical psychologists receive weekly peer supervision as well as biweekly group supervision by a specialist in clinical child psychology.

### Cool Kids Anxiety Program - Social Enhanced

The CK-E program is based on the generic Cool Kids Anxiety Program with standard CBT strategies with additional strategies specifically focusing on SAD mechanisms based on the theories of Clark and Wells [13] and Rapee and Heimberg [44]. The additional strategies in CK-E include training in task-focused attention (to reduce self-focus) and focusing on reduction of safety-seeking behaviors. In-session behavioral experiments are used to investigate the role of self-focus and safety-seeking behaviors and to reduce them. Furthermore, cost exposure and cognitive restructuring focusing on overestimation of costs, not of simply likelihood, are essential. Interpersonal exposure tasks with video feedback are used to alter adolescents’ distorted negative self-images, and post-event cognitive restructuring is used after challenging exposure tasks in order to reduce post-event rumination.

### Cool Kids Anxiety Program

The standard version of CK is based on standard CBT techniques such as cognitive restructuring and gradual exposure. Additional techniques in the CK manual include simple cool breathing (a relaxing technique) and systematic problem solving.

The therapists are instructed not to use the treatment component specifically included in the enhanced version (e.g., attention training, reduction of safety-seeking behaviors, cost exposure, video feedback, and post-event cognitive restructuring), unless they are suggested to do so by the adolescents themselves.

Therapist adherence will be assessed by independent raters on a scale developed at the Macquarie University by watching videotaped therapy sessions and coding for adherence or violations from the treatment manuals.

### Measures

Table 2 presents an overview of the included outcome measures.

**Table 2:** Overview of outcome measures, respondents, and assessment points

Measures	Respondents	Time			
		T1	T2	T3	T4
Primary outcome measure:					
ADIS-IV C/P	Y, P	●	●	●	
SPIN	Y	●	●	●	●
SCAS	Y, P	●	●	●	●
Secondary outcome measures:					
CALIS	Y, P	●	●	●	●
S-MFQ	Y, P	●	●	●	
NEQ	Y, P		●		
CHU-9D	Y	●	●	●	●
Other measures:					
Background information	P	●	●	●	●
DASS	Y, P	●	●	●	
CEQ	Y, P	●^a^			
ESQ	Y, P		●		

Abbreviations: *Y* youth, *P* parent, *T* time, *ADIS-IV C/P* Anxiety Disorders Interview Schedule for DSM-IV, Child and Parent Version, *SPIN* Social Phobia Inventory, *SCAS* Spence Children’s Anxiety Scale, *CALIS* Child Anxiety Life Interference Scale, *S-MFQ* Short version of the Mood and Feelings Questionnaire, *NEQ* Negative Effects Questionnaire, *CHU-9D* Child Health Utility 9D, *DASS* Depression Anxiety Stress Scale, *CEQ* Credibility/Expectancy Questionnaire, *ESQ* Experience of Service Questionnaire

^a^CEQ is completed by youth and parents after session 1

### Primary outcome measures

#### Anxiety Disorders Interview Schedule for DSM-IV, Child and Parent Version (ADIS-IV C/P) [52]

ADIS-IV C/P is a semi-structured diagnostic interview conducted with youth and parents separately to assess the diagnostic criteria for anxiety disorders in accordance with the Diagnostic and Statistical Manual of Mental Disorders (DSM)-IV as well as other disorders often comorbid with anxiety (e.g., depression and ADHD). Severity of the diagnosis is measured on a 9-point Likert scale ranging from “not disturbed at all” to “severely disturbed” (0–8). CSR scores of 4 or greater indicate a clinical diagnosis. Separate CSRs are made by youths, parents, and the clinician, but only the CSRs provided by the clinician are used. The most impairing diagnosis, as assessed by the clinician, is considered as the primary diagnosis. Both concurrent validity and test-retest reliability have been established for the anxiety disorder section of ADIS-C [53, 61]. The ADIS interviews are conducted by clinical psychologists and trained graduate psychology students blinded to treatment condition.

#### Social Phobia Inventory (SPIN) [14]

SPIN is a questionnaire used for measuring youths’ self-rated SAD symptoms. It includes 17 items covering SAD symptoms of fear, avoidance, and physiological/bodily reactions (trembling, blushing, heart palpitations, and sweating). The adolescents are asked to which degree they have been bothered by these symptoms during the preceding week. Each item is rated on a 5-point Likert scale (0–4). Higher scores indicate higher degree of distress regarding the symptom. The SPIN has been found to have good internal consistency, test-retest reliability, and convergent and divergent validity [3, 14]. SPIN has demonstrated good psychometric properties for assessing youth SAD [41, 43, 59].

#### Spence Children’s Anxiety Scale (SCAS and SCAS-P) [55]

SCAS and SCAS-P are used to measure adolescent- and parent-rated anxiety symptoms. The adolescent version contains 44 items (including six positive filler items), and the parent version contains 38 items. Items are rated on a 4-point Likert scale (0–3). Higher scores indicate higher levels of anxiety. It consists of six subscales reflecting symptoms specifically related to social phobia, panic disorder and agoraphobia, generalized anxiety disorder, obsessive–compulsive disorder, separation anxiety disorder, and fear of physical injury. Each subscale is scored separately; the subscales are then added together for a total score reflecting overall anxiety symptoms. The Danish version of the SCAS has shown good to excellent internal consistency in clinical and non-clinical samples and good test-retest reliability in a non-clinical sample [4].

### Secondary outcome measures

#### Child Anxiety Life Interference Scale (CALIS) [34]

CALIS is used to measure the impact of youth anxiety on various areas of life functioning, including friends, school, extracurricular activities, and family. The impact is evaluated separately by adolescents (9 items) and their parents (16 items). Items are evaluated on a 5-point Likert scale (0–4). Higher scores indicate a higher degree of life interference. CALIS has shown satisfactory internal consistency and moderate test-retest reliability [34].

#### Short version of the Mood and Feelings Questionnaire (S-MFQ) [19]

S-MFQ is used in the present study to measure depressive symptoms within the last 2 weeks. The symptoms are evaluated independently by adolescents and parents. The short version includes 13 items rated on a 3-point Likert scale. The Danish version of the full MFQ has shown good psychometric properties [22].

#### Negative Effects Questionnaire (NEQ) [48]

NEQ is a self-administered measure of negative effects of psychological treatment. For this study we slightly adjusted the language of NEQ to suit the adolescent age group. Both adolescents and parents are to complete the NEQ, which consists of three parts. The first part asks if specific negative events had occurred during treatment (yes/no). The second part is scored only if the negative event occurred; if so, participants rate how negative the effect was on a 4-point Likert scale, ranging from “not at all” to “extremely” (0–4). Finally, they attribute the negative effect to either “the treatment they receive” (1) or “other circumstances” (0). NEQ shows acceptable psychometric properties [48].

#### Child Health Utility 9D (CHU-9D) [57]

CHU-9D is a measure of health-related quality of life specifically developed for youth. In this study CHU-9D is rated by the adolescents. The scale consists of nine dimensions: worry, sadness, tiredness, pain, annoyance, school work, daily routine, sleep, and activities, each with one item rated from 1 to 5. CHU-9D has been validated in Australia and Great Britain indicating good psychometric properties [11, 23].

### Other measures

#### Background information

Parents will complete a background questionnaire before treatment. This questionnaire includes information regarding the parents’ mental and physical health, the adolescent’s mental and physical health, family demographics, household income and parents’ level of education, adolescent’s previous and/or ongoing treatment, and school absenteeism.

#### Depression Anxiety Stress Scale (DASS) [32]

DASS is a measure with three subscales on anxiety, depression, and stress. In this study DASS is used by parents to rate their own symptoms. The scale has 42 items, each rated on a 4-point Likert scale (0–3) with higher scores indicating a higher degree of distress. DASS has shown good psychometric properties [2].

#### Credibility/Expectancy Questionnaire (CEQ) [20]

CEQ is a self-rated measure addressing the participants’ expectancy and credibility about the treatment. Both adolescents and parents complete the questionnaire. CEQ consists of six items, three items regarding credibility and three items regarding expectancy. To meet the requirements of the original version of CEQ, items 1, 2, 3, and 5 are scored on a 9-point scale, while items 4 and 6 are scored on an 11-point scale. Items 4 and 6 are recoded to a 9-point scale before summarizing the scales, as has been done in other studies, e.g., Smeets et al. [54]. Both subscales have shown good test-retest reliability and high internal consistency [20].

#### Experience of Service Questionnaire (ESQ)

ESQ (Experience of Service Questionnaire) measures participants' satisfaction with the intervention [6]. There are separate items for parents (10 items) and adolescents (7 items). ESQ includes both positive and negative statements, and items are rated on a 3-point Likert scale (0–2). ESQ includes open questions with the possibility for qualitative feedback.

### Measures for mediational analysis

Table 3 presents an overview of measures for mediational analysis.

**Table 3:** Overview of measures for mediational analysis, respondents, and assessment points

Measures	Respondent	Time					
		Pre	S1	S4	S7	S10	Post
SAFE	Y	●					●
CATS	Y	●					●
Mini-SPIN	Y		●	●	●	●	
FAQ	Y		●	●	●	●	
PTQ	Y		●	●	●	●	

Abbreviations: *Y* youth, *P* parent, *S* session, *SAFE* Subtle Avoidance Frequency Examination, *CATS* Children’s Automatic Thoughts Scale, *Mini-SPIN* Mini version of the Social Phobia Inventory, *FAQ* Focus of Attention Questionnaire, *PTQ* Post-event version of the Thought Questionnaire

### Pre-/post-measures

#### Subtle Avoidance Frequency Examination (SAFE) [17]

SAFE is a self-rated measure designed to assess safety behaviors. In this study adolescents complete the questionnaire. SAFE is designed to incorporate active safety behaviors, subtle restriction of behavior, and behaviors aimed at avoiding or concealing physical symptoms. SAFE consists of 32 items ranging on a 5-point Likert scale (0–4), with higher scores indicating a higher degree of safety-seeking behaviors. SAFE has shown good psychometric properties [17, 58]. SAFE was originally designed for adults but can be used reliably and validly to assess safety behaviors in adolescents [58].

#### Children’s Automatic Thoughts Scale (CATS) [50]

CATS, which measures the range of a youth’s self-reported negative self-statements, is completed by the adolescents. CATS includes four subscales relating to automatic thoughts on social threat, personal failure, hostility, and physical threat. The full scale has 32 items, each scored on a 5-point Likert scale (0–4), with higher scores indicating higher degrees of negative automatic thoughts. Only two subscales with 20 items are included in the present study, those on thoughts related to social threat and personal failure. The subscales have shown correlations with self-rated SAD in a prior study [38]. CATS has demonstrated good psychometric properties [38, 49, 50].

### Repeated measures

#### Mini version of the Social Phobia Inventory (Mini-SPIN) [15]

Mini-SPIN is developed as a brief screening instrument for SAD but can also serve as a repeated outcome measurement. Mini-SPIN is completed by the adolescents, and it includes three specific items from the original SPIN (“Fear of embarrassment causes me to avoid doing things or speaking to people”; “I avoid activities in which I am the center of attention”; and “Being embarrassed or looking stupid is among my worst fears”). Each item is rated on a 5-point Likert scale (0–4). Higher scores indicate higher degree of distress regarding the symptom. Mini-SPIN has demonstrated high sensitivity and specificity [51] and good psychometric properties for assessing adolescents with SAD [42].

#### Focus of Attention Questionnaire (FAQ) [62]

FAQ is a self-rated measure of focus of attention and is completed by the adolescents themselves. FAQ is a 10-item scale including two 5-item subscales, self-focused attention and external-focused attention. All items are rated on a 5-point Likert scale ranging from 1 to 5 (1 = not at all”, 5 = “totally”). Subscale scores are calculated by averaging the five items. A higher score on the two subscales indicates a higher degree of self-focused attention or external focus, respectively. High internal consistency has been reported [63].

#### Post-event version of the Thoughts Questionnaire (PTQ) [21]

PTQ is a self-rated measure to assess the degree of post-event processing. Adolescents complete the PTQ. Consistent with prior research [40, 60], only the 15 negatively worded items of the PTQ are used in this study. All items are rated on a 5-point Likert scale (0–4). Higher scores indicate a higher degree of post-event rumination. Studies have shown excellent internal consistency [1, 40, 60].

### Economic evaluation

Cost–utility analyses will be performed by use of the CHU-9D, which was designed to determine how health affects children’s lives, and is rated by the youth. The CHU-9D is a generic preference-based measure of health-related quality of life, designed for the estimation of quality-adjusted life years (QALYs) for economic evaluation of healthcare treatment.

### Administration of measures

SPIN, SCAS, CALIS, and CHU-9D are assessed pre- and post-intervention and at the 3months and 1 year follow-ups. ADIS-IV C/P, S-MFQ, DASS, SAFE, and CATS are assessed at pre- and post-intervention and at the 3 months follow-up. NEQ and ESQ are assessed post-treatment. CEQ is completed after session 1. Mini-SPIN is assessed before sessions 1, 4, 7, and 10. FAQ and PTQ are assessed at sessions 1, 4, 7, and 10.

All questionnaires are administered electronically.

**Fig. 2:**
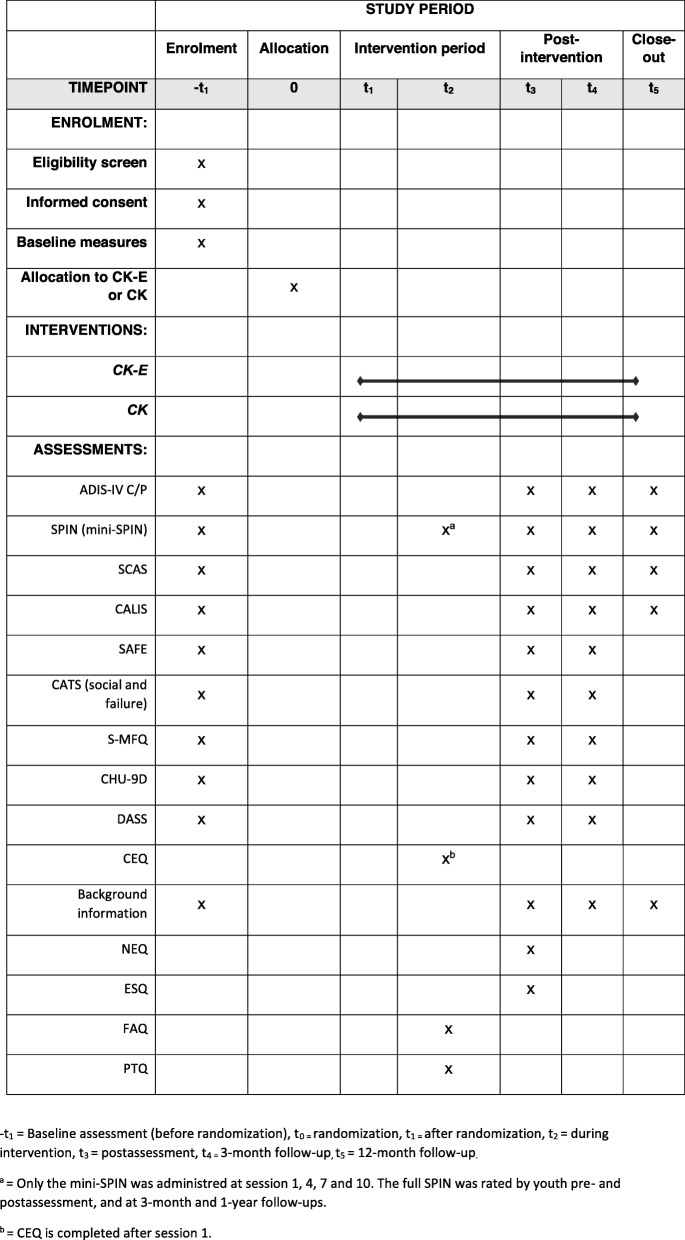
SPIRIT diagram showing schedule of enrollment, allocation, interventions, and assessments. –t_1_ = baseline assessment (before randomization), 0 (t_0_) = randomization, t_1_ = after randomization, t_2_ = during intervention, t_3_ = post-assessment, t_4_ = 3 months follow-up, t_5_ = 1 year follow-up. ^a^Only the mini-SPIN was administered at sessions 1, 4, 7, and 10. The full SPIN was rated by youth pre- and post-assessment, and at 3 months and 1 year follow-ups. ^b^CEQ is completed after session 1

Figure 2 shows an overview for the completed Standard Protocol Items: Recommendations for Interventional Trials (SPIRIT) figure. The SPIRIT checklist is provided as Additional file 1.

### Statistical analysis

The targeted sample size with 47 adolescents per treatment condition (94 in total) will provide an acceptable statistical power (0.81; α = 0.05, two-tailed) to find an effect size of *d =* 0.65. Since there is no prior comparable study, it is difficult to estimate an expected effect size. In a study of the Cool Kids Anxiety Program at our clinic, youths with SAD achieved an effect size (*d*) of – 1.0 compared to that of other anxiety disorders (Arendt et al., 2015).

Multilevel linear models (MLMs) are used to compare groups over time (T1, T2, T3, T4) for all outcome variables. MLMs tolerate missing data and therefore do not compromise statistical power unnecessarily. All MLMs are based on intention-to-treat samples. All MLMs are estimated with the maximum likelihood method.

Two sorts of mediational analyses are planned to be conducted: formal mediational analyses with mediators measured pre- and post-therapy by use of the product-of-coefficients method [35] and time-lagged analyses of change with repeated measures within both groups [18].

## Discussion

Developing effective treatment for youth SAD is important because of the high number of adolescents suffering from this disorder. Youth SAD is associated with extensive distress and long-term impairment, which underpins the importance of thorough research. Recent studies indicate that adolescents with SAD have poorer outcomes following generic treatment compared to adolescents struggling with other anxiety disorders [26, 31, 33, 46].

To the best of our knowledge, no prior studies have compared generic and diagnosis-specific CBT for SAD with the same treatment format. An aforementioned study indicates that diagnosis-specific treatment for youth SAD may be more effective than generic treatment [27]. The present study compares a diagnosis-specific G-CBT program with a generic G-CBT program for SAD. The present study will provide more information about the efficacy of diagnosis-specific G-CBT for youth SAD.

### Trial status

A feasibility study with 13 youth (aged 12–16 years) with a SAD diagnosis was conducted in the fall of 2017. Participants were assigned to three treatment groups, all of which used the CK-E approach. The study showed low dropout and high scores on satisfaction and indicated that the manual was adaptable to a group format. Based on the experiences from the feasibility study, some procedures were revised before the RCT. Inclusion of participants to the RCT started in February 2019 and is expected to be finished by September 2021.

## Supplementary information

**Additional file 1.** SPIRIT 2013 checklist: recommended items to address in a clinical trial protocol and related documents.

## Data Availability

Not applicable.

## Abbreviations

ADIS-IV C/P: Anxiety Disorders Interview Schedule for DSM-IV, Child and Parent Version
CALIS: Child Anxiety Life Interference Scale
CATS: Children’s Automatic Thoughts Scale.
CBT: Cognitive behavioral therapy
CEBU: Center for Psychological Treatment of Children and Adolescents
CEQ: Credibility/Expectancy Questionnaire
CHU-9D: Child Health Utility 9D
CK: Cool Kids Anxiety Program
CK-E: Cool Kids Anxiety Program - Social Enhanced
CSR: Clinical Severity Rating
DASS: Depression Anxiety Stress Scale
ESQ: Experience of Service Questionnaire
FAQ: Focus of Attention Questionnaire
Mini-SPIN: Mini version of the Social Phobia Inventory
NEQ: Negative Effects Questionnaire
PT: Post-event version of the Thoughts Questionnaire
RCT: Randomized controlled trial
SAD: Social anxiety disorder
SAFE: Subtle Avoidance Frequency Examination
SCAS: Spence Children’s Anxiety Scale
S-MFQ: Short version of the Mood and Feelings Questionnaire
SPIN: Social Phobia Inventory
